# Noninvasive Brain Stimulation and Personal Identity: Ethical Considerations

**DOI:** 10.3389/fnhum.2017.00281

**Published:** 2017-06-07

**Authors:** Jonathan Iwry, David B. Yaden, Andrew B. Newberg

**Affiliations:** ^1^Department of Psychology, University of PennsylvaniaPhiladelphia, PA, United States; ^2^Myrna Brind Center for Integrative Medicine, Thomas Jefferson UniversityPhiladelphia, PA, United States

**Keywords:** noninvasive brain stimulation, neuroethics, identity, informed consent, autonomy

## Abstract

As noninvasive brain stimulation (NIBS) technology advances, these methods may become increasingly capable of influencing complex networks of mental functioning. We suggest that these might include cognitive and affective processes underlying personality and belief systems, which would raise important questions concerning personal identity and autonomy. We give particular attention to the relationship between personal identity and belief, emphasizing the importance of respecting users' personal values. We posit that research participants and patients should be encouraged to take an active approach to considering the personal implications of altering their own cognition, particularly in cases of neurocognitive “enhancement.” We suggest that efforts to encourage careful consideration through the informed consent process would contribute usefully to studies and treatments that use NIBS.

## Introduction

Noninvasive brain stimulation (NIBS) increasingly enables researchers and clinicians to safely and effectively alter brain function. Whether used for therapeutic purposes or for purposes of neurocognitive “enhancement,” these and other forms of neurological technology are the subject of considerable scrutiny. Their scope and relative novelty raise possibilities for improved cognition and general well-being, but also a host of concerns, typically involving questions of justice, fairness, and, for some, threats to certain cultural norms or values (Farah et al., 2004; Sandel, 2009).

Among these concerns is the potential impact of NIBS and related technologies on personal identity. Could lower-level cognitive alterations have unexpected effects on higher-level aspects of cognition? If so, the use of NIBS, while medically safe, might pose ethical challenges regarding freedom of personal belief—or, more broadly, what is sometimes called “cognitive liberty” (Sententia, 2004).

This article examines the potential effects of NIBS on personal identity and their implications for prospective users. We argue that NIBS might be capable of influencing cognitive processes, and perhaps even personally meaningful belief systems, in ways that could affect patients' pursuit of well-being and attitudes toward their own identities. Given this possibility, it is worth considering how to encourage users of NIBS to adequately address its personal implications, whether in therapeutic or enhancement settings. We suggest that measures to encourage active self-determination could promote responsible use of NIBS and contribute to its potential as an instrument of medical and psychiatric welfare.

## Potential applications and safety of NIBS

“NIBS” refers to technologies that affect neural activity without physical penetration. The two main forms of NIBS currently in use are trans-cranial magnetic stimulation (TMS) and trans-cranial direct current stimulation (tDCS). TMS uses a magnetic field to create neuronal action potentials inside the brain, sometimes using repeated pulses to create a longer-term effect (rTMS) (Maeda and Pascual-Leone, 2003). tDCS affects the polarity of neurons' resting membrane potential in targeted regions (Nitsche and Paulus, 2000).

The efficacy of NIBS techniques is being tested for a range of applications. Promising results with treating depression (George et al., 2010) have led the U.S. Food & Drug Administration to license its use as an antidepressant therapy, with other therapeutic applications in the approval pipeline. Some studies indicate a potential for treating symptoms of Parkinson's disease (Fregni et al., 2005a; Boggio et al., 2006) and restoring speech and motor control to stroke victims (Webster et al., 2006; Blesneag et al., 2015; Wessel et al., 2015).

The potential benefits of NIBS extend even beyond therapy for specific disorders. Studies have explored the capacity for NIBS to improve visual and spatial processing (Hilgetag et al., 2001). Results have been especially promising for attempts to enhance working memory and learning speed with respect to motor and language tasks (Kim et al., 2004; Fregni et al., 2005b; Flöel et al., 2008; De Vries et al., 2010). Some researchers have speculated that these technologies will soon offer a form of enhancement or “neurocosmetics” that can improve general cognition, susceptibility to positive emotion, and even artistic ability among those who are already healthy (Hamilton et al., 2011).

The most commonly reported risks of NIBS are relatively minor, though it is possible that side effects have been under-reported (Brunoni et al., 2011). Headaches and local pain are the most common effects of TMS, with meta-analyses reporting rates between 28 and 40% (Machii et al., 2006; Loo et al., 2008). Other patients report various forms of discomfort from being immobilized for long periods of time, and even nausea in some cases (Satow et al., 2002; Machii et al., 2006). Machinery used in TMS is loud enough to affect hearing temporarily and even, in one case, permanently (Loo et al., 2001; Zangen et al., 2005) resulting in the widespread use of earplugs. Minor burns to the scalp and twitches from stimulated scalp muscles have also been reported (Roth et al., 1992; O'Reardon et al., 2007; Rossi et al., 2009).

Psychiatric risks also exist, though they appear to be very rare. There have been occasional reports of mania during rTMS treatment of the left PFC for depression and bipolar disorder (Garcia-Toro, 1999; Dolberg et al., 2001; Xia et al., 2008), as well as reports of delusions and psychotic symptoms (Ella et al., 2002; Janicak et al., 2008). It is unclear whether those symptoms occurred more frequently than average for the illnesses being treated (Rossi et al., 2009). There is also a risk of seizures, though they have also been rare; seizures associated with TMS and rTMS, for instance, date largely to the period before safety guidelines were established for its use, and of those that have occurred since 1998, most have involved subjects on medications with pro-epileptogenic effects (Rossi et al., 2009). One highly vulnerable pediatric patient reportedly experienced a seizure during tDCS (Ekiki, 2015).

## Concerns regarding NIBS and its popular appeal

Some have suggested that the use of NIBS, particularly for enhancement, calls for greater caution. This would include more careful evaluation of the use of NIBS on children, given the higher stakes and uncertain future effects for brains still undergoing rapid and formative development (Cohen Kadosh et al., 2012). In addition, Davis and van Koningsbruggen (2013) point out that “noninvasive” is something of a misnomer, since electrical currents are directed into the brain with the purpose of affecting cortical functioning. Any direct interference with neural activity, even beneficial, might be described more accurately as “minimally invasive” (Fitz and Reiner, 2015). Of course, the fact that NIBS is not distressing during treatment does not mean it cannot have distressing implications for the patient, and the fact that it does not break the skin does not mean, figuratively speaking, that it cannot cross other important personal boundaries.

Despite researchers' discussion of and explicit warnings against unsupervised use of NIBS (Wurzman et al., 2016), brain stimulation products are already commercially available, and online websites offer instructions for building do-it-yourself devices. Without proper guidance or information, NIBS could be conducted recklessly or even in conjunction with other means of cognitive alteration, with unknown and potentially harmful effects.

Such issues have already been raised in the psychopharmacological realm. Surveys suggest that many college students use Ritalin and other “study drugs” without prescriptions, hoping to improve their attention and memory even without having been diagnosed with a disorder (Smith and Farah, 2011; Farah, 2015b). That such medications are easily accessible and fairly widespread across college campuses might encourage students to experiment with those medications without considering the risks and unknowns of reckless use (or, if communal pressure is strong enough, in spite of them). Neuromodulation acts on different mechanisms, and thus comes with different unknowns, than those of psychopharmacology. Yet the rise in study-drug abuse would seem to offer a warning that public excitement about enhancement might impede careful and reflective decision-making, especially when trying to boost cognitive functions that are already at healthy levels or when applying treatments in conditions in which they have not been demonstrated to be effective.

In some respects, NIBS might be even more prone to deceptive advertisement. That this class of technologies is advertised as “noninvasive” may encourage unchecked enthusiasm about its applications. As an “external” rather than “internal” form of stimulation (Cohen Kadosh et al., 2012), NIBS might lack much of the public stigma associated with psychoactive substances, leading some users to assume without justification that its effects are not as strong.

The use of any sort of cognitive enhancement without proper consideration of the personal and environmental context of its use could lead users to undermine their own attempts to improve cognition or general well-being. On one hand, the public appears interested in questions about cognitive enhancement and sensitive to crucial considerations (Fitz et al., 2014; Schelle et al., 2014), but the potential for laypeople to be deceived by fallacious appeals to neuroscience (Weisberg et al., 2008; Wurzman et al., 2017) raises concerns about their ability to relate scientific findings and explanations to moral questions in both public and private life. Patients might not be appropriately skeptical of unwarranted or exaggerated marketing statements; even in medical environments, they might underestimate the relevance of certain personal considerations to their decisions about treatment.

## NIBS and personal identity

The convenience and relative safety of NIBS make it uniquely appealing. However, like deep brain stimulation and psychopharmacological intervention, NIBS manipulates neurobiological and cognitive processes (Yaden et al., 2015). The various treatments differ significantly in their methods of intervention, yet all three seek to influence mental functioning through measures over which the patient lacks direct control, raising concerns for patients who value certain aspects of their personal identity. This has already been acknowledged with regard to deep brain stimulation (Schermer, 2011; Maslen et al., 2015) and psychopharmacology (Elliott, 2004; Kramer, 2004), and, as discussed below, also merits consideration in the case of NIBS.

Because of its potential to influence emotion, attention, reasoning, and social behavior (Hamilton et al., 2011), NIBS has implications for personal identity that are no less serious than those of any other method that modulates cognitive processes. Our view of our emotional and behavioral attributes is a major part of how we understand and relate to ourselves. Individuals who suffer from attention and behavior disorders, for example, often struggle to reconcile their intrusive thoughts and emotions with their own conceptions of their personalities, and they are attuned to the effects of psychopharmacological changes in temperament on their relationships with others (Bolt and Schermer, 2009; Maslen et al., 2015). These issues are a basic part of the individual's decision to turn to medication for treatment, and they are no less present when deciding whether to use neuromodulation instead.

There is also the problem of unintended consequences. Attempts at enhancement may be limited by underlying neurocognitive constraints, meaning that boosting certain traits or abilities might come with tradeoffs for others (Iuculano and Cohen Kadosh, 2013). Effects of identical types of stimulation on cognitive performance can also vary significantly between individuals (Sarkar et al., 2014). Because cognitive processes span vast networks of brain regions (Kato et al., 2009), and because much remains to be understood about the processes affected by NIBS (Rajapakse and Kirton, 2013), targeting brain structures to alter cognition could yield unwanted side effects, mitigate the desired outcome, or even result in paradoxical responses. (It is worth clarifying that the issue of unintended consequences is not unique to NIBS. Indeed, the more spatially localized methods of intervention used in neuromodulation may lack the more diffuse effects of pharmacological agents. We do note, however, that off-target stimulation is a concern when using NIBS; Davis et al., 2013).

### Belief and identity

Is it possible for lower-level cognitive alterations to have pervasive effects on much higher-level processes, such as those involving belief? Of particular interest here are beliefs (or similar states) concerning the individual's “deep values” (Veatch, 1995). These are attitudes, beliefs, and commitments of particular importance to the individual that relate to central aspects of their worldview and their conception of their identity. These might include social attitudes, moral beliefs, religious, and political inclinations—beliefs that govern daily behavior and give meaning to our pursuits and experiences.

The apparent influence of automatic processes on belief and complex states of reflection is a topic of interest to cognitive scientists and philosophers. Some have called for an expanded topography of mental states related to belief, arguing that the nature and role of belief, even in its common conception as a mental description of a state of affairs, would be better understood within a broader context of experience and behavior (Schwitzgebel, 2001; Gendler, 2008). Much of this research emphasizes that belief operates on physical systems, the components of which have yet to be clearly understood and seem to overlap in diverse ways (Damasio, 1999). These may include close linkages between perception and emotional and sensory imagination (Araujo et al., 2013). Reactions to intense or threatening stimuli often appear to qualify as something akin to belief and trigger rapid emotional and behavioral responses (Rudrauf et al., 2008).

Automatic processes interact and even conflict with beliefs and deliberate reasoning in bringing about behavior. For example, it has been shown that many judgments rely more on automatic processes than conscious reflection (Kahneman, 2011). Many of our attitudes toward moral decisions, even when formalized as clear-cut propositions, can shift in response to differences in presentation (Tversky and Kahneman, 1981; Taber and Lodge, 2006). Considerable research has been devoted to the relationship between and apparent influence of disgust and other forms of emotional processing on political ideology (Amodio et al., 2007; Ahn et al., 2014; Hibbing et al., 2014). Moral intuitionists argue that moral reasoning is almost completely governed by these processes, and that beliefs and reasons arise only afterwards to support intuitive reactions (Wegner and Bargh, 1998; Haidt, 2012).

Many promising findings for NIBS involve alterations to complex cognitive processes involving attention, memory, and judgment that could subtly influence patients' beliefs. Affecting the mechanisms of belief, however indirectly, without patients' awareness or ability to mediate the effects could impinge on their values. NIBS has already demonstrated the potential to temporarily shift political orientation by increasing activity in the DLPFC (Chawke and Kanai, 2015), which is closely implicated in regulation of political attitudes (Kato et al., 2009).

These interactions between automatic processes and belief might also influence the ways that patients interpret personally meaningful experiences. Research on religious and spiritual states appears to point in this direction (Newberg et al., 2001, 2003; Urgesi et al., 2010; Yaden et al., 2017a,b; also see Yaden et al., 2016). Crescentini et al. (2014) were able to alter participants' degrees of implicit spiritual or non-spiritual identification using NIBS. For many, these temporary changes might be more akin to changes of perspective than literal changes in belief, but their effects on behavior and personal outlook suggest that they can play a significant role in patients' worldviews and influence deeply held beliefs and values.

### Challenges to autonomy

The risk that NIBS might affect central aspects of a patient's identity, including their values and belief system, raises additional concerns about their ability to preserve their “mental self-determination” (Bublitz and Merkel, 2014). If an individual holds a conception of organic personal continuity that gives coherence to their sense of self, then undergoing any kind of intervention with uncertain consequences for their thought processes could threaten that notion of integrity. Having undergone neuromodulation, they might come to worry that they have surrendered their autonomy over certain personally significant or defining mental characteristics (that they might have, as it were, estranged themselves from themselves). In addition to the risk of psychological distress, this could pose a challenge for practitioners who are concerned about how best to respect their patients' values.

## Autonomy as a guiding principle

Although unforeseen alterations to personal identity raise important questions for those considering NIBS, these become less troubling if users can evaluate and resolve them for themselves while deciding whether to undergo treatment. It would generally be of benefit to patients—and consistent with general medical practices—to emphasize individual freedom of decision-making. Recognizing the risk that users would too often pursue treatment without considering the implications for their identity, we might look for relatively unobtrusive ways of helping to make a more informed decision.

In strengthening autonomy of the patient in deciding whether to use NIBS, we might decide to prioritize active self-determination over less rigorous exercise of personal freedom. Much has been written about what counts as a legitimate autonomous judgment (Farah, 2015a); one appealing position by Savulescu (1994) is that a desire is “rationally autonomous” when it reflects judgments that are adequately informed, well-reasoned, and based on a realistic imagination of the results of acting on that desire. When individuals appear to make decisions bearing considerable risk, out of either inattentiveness or impatience, we often consider it wise to encourage them to pause and reflect rationally on their situations.

If there is indeed a risk that patients will be unprepared for the personal implications of NIBS, this adds an additional mental health aspect to their decisional calculus. It may then be in patients' interest for practitioners to help them consider the implications for their notion of selfhood and, if they choose the treatment, prepare to interpret those implications in a way that is both mentally healthy and consistent with their broader set of personal values.

## Possible directions for informed consent

Due to the potential for bias and situation to unduly influence judgment, and due to the conceptual difficulties inherent in reflecting on possible implications for personal identity, added measures associated with choice architecture and the informed consent process might benefit participants and patients in exercising their autonomy in deciding whether to use NIBS. These would allow the informed consent process to serve as a sort of checkpoint for the patient, giving them the resources to make sure that they are well-informed. The goal, in a sense, would be to encourage them to go beyond mere “consent” and take an active stance toward considering critical aspects of their well-being (Veatch, 1995).

Informed consent documents serve partly to protect medical institutions against liability, but also are intended to protect the well-being of the patient. Arguably, documents addressing the practical hazards of NIBS already do a considerable amount to serve that second goal, though perhaps even more could be done to address less tangible but no less consequential concerns for the patient's long-term ability to flourish. Adding an “identity and values” disclosure, for instance, would ensure that the patient has explicitly considered implications for their identity and other, more abstract, yet equally significant personal concerns.

Although informed consent waivers are already a necessary part of the process, they might not be sufficient. Given that individuals often fail to read or meaningfully absorb the content of these waivers, users would almost certainly benefit from an opportunity to discuss personal concerns and ask detailed questions in private conversation with psychiatrists and medical experts (Flory and Emanuel, 2004). To that end, some sort of counseling might be offered in advance of treatment to ensure that patients have reflected on any related issues of serious importance to them and can incorporate those concerns into their decisions over NIBS treatment. It is increasingly common for clinicians to employ some form of what some call “shared decision-making,” an approach to informed consent that recognizes that individuals often benefit from actively discussing options with clinicians, and that doing so improves the quality of their decisions while respecting their general autonomy (King and Moulton, 2006; Elwyn et al., 2012). This approach might usefully be applied to the informed consent process for NIBS, as well. Prospective users of NIBS would be better served in making decisions that reflect their long-term interests if they could explore the personal implications of neuromodulation in a setting that emphasized careful and explicit communication, open expression and shared evaluation of the patient's preferences, and strong patient-clinician rapport. Of course, additional complexities and the need for more stringent requirements in the consent/assent process must be considered in cases of NIBS use on children (Davis, 2014; Maslen et al., 2014).

Although the so-called “therapy-enhancement distinction” (Farah, 2015a) can sometimes be ambiguous (Earp et al., 2014), perhaps the potential impact on personal identity should be weighed more heavily when treatment is intended for enhancement rather than therapy. Patients seeking to treat Parkinson's or depression might be less concerned about comparatively subtle effects on their identities, let alone abstract notions of personal autonomy. However, a healthy individual using NIBS simply for purposes of enhancement—say, improving their memory—could be taking a risk that is harder to justify. Accordingly, additional counseling might be required, or at least encouraged, for treatments intended to make the patient “better than well” (Elliott and Chambers, 2004).

## Conclusion

The applications of brain stimulation methods labeled “noninvasive” might well-continue to expand in the future. Given that certain aspects of personal identity, or the freedom to decide one's own identity, might someday fall within the reach of such methods, we would be well-served by taking a conservative approach to cognitive alteration while seeking to protect cognitive features traditionally considered to be valuable components of personhood. In the meantime, the process of deciding whether to use NIBS could be structured to more effectively emphasize nonmedical considerations and encourage rational autonomy. Informed consent documents could, for example, acknowledge a range of potential personal implications of undergoing NIBS. Counseling could also be offered to expose participants and patients to useful information and to encourage careful consideration of their interests and options. Respect for individuals' personal autonomy and values constitutes an important aspect of many therapeutic, enhancement, and research procedures–NIBS, while “noninvasive,” ought to be included among them. Improvements to informed consent might not settle the fundamental debates concerning the ethics of personal identity, but they might well-complement those debates and prove to be a step in the right direction.

## Author contributions

JI and AN: writing and editing. DY: conception, writing, and editing.

### Conflict of interest statement

The authors declare that the research was conducted in the absence of any commercial or financial relationships that could be construed as a potential conflict of interest.
